# Flavivirus: From Structure to Therapeutics Development

**DOI:** 10.3390/life11070615

**Published:** 2021-06-25

**Authors:** Rong Zhao, Meiyue Wang, Jing Cao, Jing Shen, Xin Zhou, Deping Wang, Jimin Cao

**Affiliations:** 1 Key Laboratory of Cellular Physiology, Ministry of Education, Shanxi Medical University, Taiyuan 030001, China; zhaorong@b.sxmu.edu.cn (R.Z.); wangmeiyue0808@b.sxmu.edu.cn (M.W.); caojing@b.sxmu.edu.cn (J.C.); shenjing@sxmu.edu.cn (J.S.); 2 Department of Physiology, Shanxi Medical University, Taiyuan 030001, China; 3 Department of Medical Imaging, Shanxi Medical University, Taiyuan 030001, China; xzhou@sxmu.edu.cn

**Keywords:** flavivirus, protein structure, vaccine, drug target, immunology, epidemics

## Abstract

Flaviviruses are still a hidden threat to global human safety, as we are reminded by recent reports of dengue virus infections in Singapore and African-lineage-like Zika virus infections in Brazil. Therapeutic drugs or vaccines for flavivirus infections are in urgent need but are not well developed. The Flaviviridae family comprises a large group of enveloped viruses with a single-strand RNA genome of positive polarity. The genome of flavivirus encodes ten proteins, and each of them plays a different and important role in viral infection. In this review, we briefly summarized the major information of flavivirus and further introduced some strategies for the design and development of vaccines and anti-flavivirus compound drugs based on the structure of the viral proteins. There is no doubt that in the past few years, studies of antiviral drugs have achieved solid progress based on better understanding of the flavivirus biology. However, currently, there are no fully effective antiviral drugs or vaccines for most flaviviruses. We hope that this review may provide useful information for future development of anti-flavivirus drugs and vaccines.

## 1. General Information of Flavivirus and the Infected Diseases

Flavivirus is a genus of viruses that have infected people in most parts of the world. Flavivirus belongs to the positive-sense single-stranded RNA viruses with a genome size of approximately 11 kb [1]. This class includes more than 70 types of small envelope viruses, including the most recognized dengue virus (DENV), Zika virus (ZIKV), West Nile virus (WNV), yellow fever virus (YFV), Japanese encephalitis virus (JEV), Tick-borne encephalitis virus (TBEV), etc. Although flaviviruses are abundant and variant, only a small percentage of them can infect humans [2,3]. These flaviviruses are neuroinvasive and neurovirulent, and can cause central nervous system (CNS) damage. Here, we listed some important members of the flavivirus genus and their clinical features after infection.

### 1.1. YFV

YFV causes yellow fever, an infectious disease also named “Blood Vomit”. YFV is transmitted by a commonly known yellow fever mosquito, Aedes aegypti. Aedes aegypti is a native African mosquito and, not coincidently, YF originates in Africa. YFV is transmitted through an urban cycle and wild species cycle. Via the slave trade, YF was introduced to America and other countries [4,5]. Given that this mosquito is highly susceptible to YFV, Aedes aegypti became the primary transmitter and has had the greatest impact on human health [6]. From the 18th century to the early 20th century, YFV has caused a major threat to humans. This repeated epidemic was discovered in North America, the Caribbean, and Europe. YFV can cause severe acute illness with nausea, vomiting, hemorrhage, jaundice, multiple organ dysfunction such as acute liver injury, and death in 20%–60% of cases [7,8]. Fortunately, an effective attenuated vaccine from a strain of YFV has been developed, which greatly assisted in controlling the epidemics of YFV and laid the foundation for developing more useful vaccines against other flaviviruses [9,10].

### 1.2. DENV

DENV is transmitted mainly by Aedes aegypti and Aedes albopictus, which are the main mosquito species responsible for DENV transmission to humans in the tropical and subtropical areas of the world [11,12]. The typical characteristics of DENV are dengue fever, dengue hemorrhagic fever, and dengue shock syndrome with high morbidity and mortality [4,12]. The disease was recorded in the middle and late 18th century [3,13]. The infected population is mainly concentrated in tropical and subtropical areas, and approximately 390 million people are infected annually [7]. In 2009, WHO classified dengue fever as an acute febrile disease. Subsequently, dengue virus is divided into four serotypes according to the antigenicity of the viral envelope protein E (DENV-1, DENV-2, DENV-3, and DENV-4), each of which is capable of causing dengue fever, dengue hemorrhagic fever, or dengue shock syndrome [14,15]. DENV infection shows a typical phenomenon of antibody-dependent enhancement (ADE). It is doubtless that ADE can cause increased disease severity [16]. Increased risk of severe disease occurs during secondary infection with a virus serotype distinct from that of prior dengue infection. This occurs by antibody dependent enhancement (ADE) of infection, wherein sub-neutralizing antibodies against the virus particles opsonize dengue virus entry via formation of immune complexes that interact with fragment crystallizable gamma receptors (FcγR) on monocytes, dendritic cells, and macrophages [16,17]. At the beginning of the 21st century, multiple cases of dengue virus concurrent infection with different serotypes have been reported frequently in many places [18]. In individual cases of infection, some patients were simultaneously infected by more than two serotypes of DENV who were skewed to more severe clinical manifestations compared to mono-infected patients [19,20]. In severe cases, because of the cross-reaction of antibodies produced by different types of DENV, patients showed symptoms such as diarrhoea, fever, severe plasma exudation, bleeding, and multiple organ damage [21,22,23]. Therefore, there is an urgent need to develop an effective vaccine containing all four DENV serotypes.

### 1.3. JEV

JEV is transmitted mainly by Culex mosquitoes. Although many flaviviruses can cause encephalitis, JEV causes particularly severe neurological manifestations. The first outbreak of the disease in Japan was noted as early as 1871. Since the first case was reported, the number of infected cases has increased year by year, and approximately 68,000 cases are found annually [24,25]. The main clinical manifestations of this disease are fever, headache, signs of neuralgia, such as kerato-toxoplasma and pseudo-facial features, while some cases can develop to encephalitis, meningitis, coma, and death. Approximately 30% of the infected patients have neurological sequelae [26,27]. JEV causes more loss of disability-adjusted life years than any other arthropod-borne virus owing to the frequent neurological sequelae of the condition.

### 1.4. WNV

WNV is also a neurotropic virus [28]. It was transmitted to human beings mainly by Culex mosquitoes [4,29]. In 1937, WNV was first isolated from a case in Uganda [30]. In 1999, the disease caused by WNV became an endemic in the USA and posed a significant threat to the health of local people. As WNV encephalitis can cause serious consequences to human health, this virus gains worldwide concern [31,32]. In addition, approximately 80% of the cases were subclinical infections [33]. Mild cases may develop myalgia, arthralgia, maculopapular rash, and other symptoms. Severe cases show neurological symptoms, such as acute flaccid paralysis, meningitis, encephalitis, and other long-term sequelae, or even death [34,35].

### 1.5. ZIKV

ZIKV is another arbovirus transmitted by mosquitoes, mainly Aedes [36]. ZIKV was first found in Rhesus monkeys in the Zika forest area of Uganda in 1947. Subsequently, three cases of human ZIKV infections in Uganda and the United Republic of Tanzania were described formally in 1952 [37]. A large-scale outbreak of ZIKV infection occurred in Brazil in 2015, approximately 1.3 million people were infected, and the disease spread rapidly in South and Central America [38,39]. It has also been reported that the transmission of this virus is through mosquito bites, sexual activity, and blood transfusion. It is worth nothing that the cases of ZIKV infections in humans reported in 2020 have reached 3600. Therefore, when dealing with SARS-CoV-2 infections, we should also pay attention to the new epidemics of ZIKV [40]. The symptoms of patients infected with ZIKV are usually mild. However, the sequelae of some ZIKV infections are serious, including severe neurological diseases. Particularly, ZIKV infection has a destructive impact on fetal development. It can also lead to abortion in pregnant women, infant microcephaly, and even the death of the infant after birth [7,34,41,42,43].

In summary, different flaviviruses have different modes of transmission, timelines of occurrence, abilities of infection, and differences in their impacts. These flaviviruses have led to large epidemics in the past, and may cause large outbreaks in the future. Therefore, it is necessary to understand the entering and replicating mechanisms of different flaviviruses in cells. Such knowledge may assist in the development of new vaccines and antiviral drugs.

## 2. The Structures and Functions of Flavivirus Proteins

**Figure 1:**
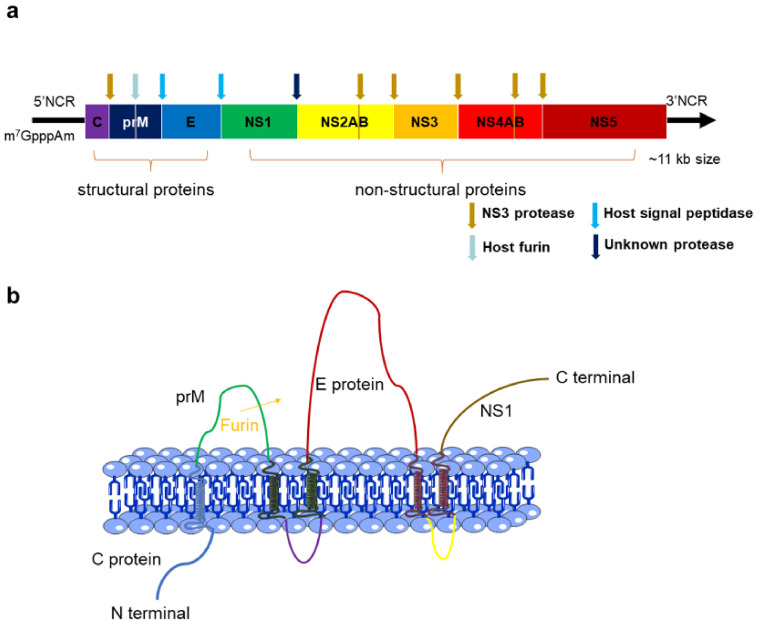
(**a**) The long open reading fame (ORF) of flavivirus genome which encodes a polyprotein. The polyprotein is cleaved into 10 proteins by proteases. Capsid protein (C protein), pre-membrane protein (prM protein), and envelope protein (E protein) are structural proteins, whereas the remaining seven are non-structural proteins. There are cleavage sites between proteins, among which the sites indicated by brown arrows can be cleaved by viral proteases, and the sites indicated by blue arrows are the cleavage sites of host proteases. In addition, there is a cap structure at the 5-terminal. (**b**) Association of the structural proteins of flavivirus. There is a furinase site between prM protein and C protein. In the process of virus transportation to the Golgi body or after virus entry into the Golgi body, the prM is cleaved by furin at this furinase site in the host cell, making the virus particles mature and infectious.

The genome of flavivirus encodes a long open reading fame flanked by a capped 5-terminal and lacks the poly tail of the 3-terminal (Figure 1a). Under the cooperation of virus and cell protease, the translated single polypeptide is cleaved into three structural proteins, namely capsid protein (C protein), pre-membrane protein (prM protein), envelope protein (E protein), and seven non-structural (NS) proteins, namely, non-structural protein 1 (NS1), NS2A, NS2B, NS3, NS4A, NS4B, and NS5 (Figure 1b). These proteins constitute the main structural components of virus particles, and participate in the key steps of the viral life cycle [2,44,45,46,47,48,49].

### 2.1. C Protein

**Figure 2:**
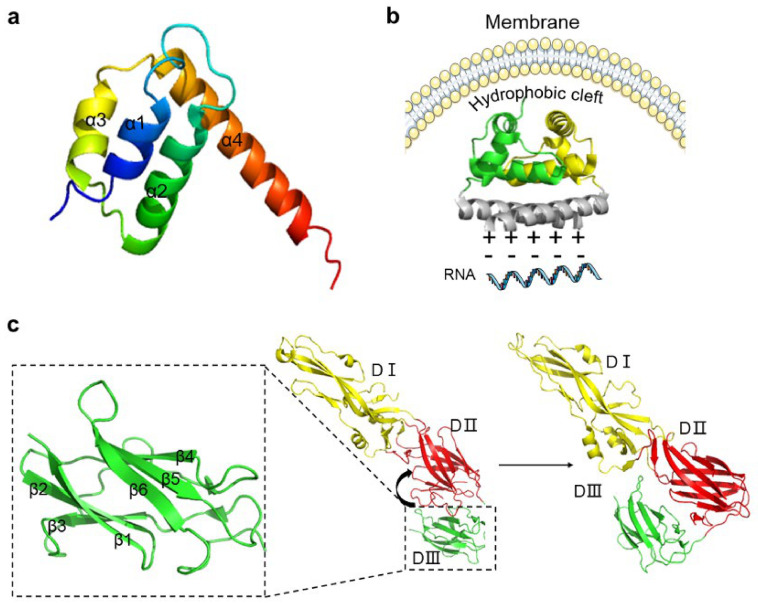
The structures of capsid protein (C protein) and envelope protein (E protein). (**a**) The structure of the dimer of C protein of dengue virus (DENV; Protein Data Bank [PDB]: 1R6R DENV). (**b**) C protein tetramer. C protein is arranged in a symmetrical form of 2:2:2, forming a hydrophobic channel in the middle of the tetramer (PDB: 1SKF West Nile virus [WNV]). (**c**) The three domains of E protein; the detail of DIII is shown in the enlargement of the framed area. There is a flexible short chain between DII and DIII; the arrow indicates the conformational change of DIII towards DII during maturation (PDB: 1TG8 DENV).

C protein is relatively small, only containing approximately 114 amino acids. A multiple-sequence alignment of mosquito-borne flavivirus capsid proteins indicates low sequence conservation. However, NMR and X-ray crystal structures of C-dimer of the flavivirus show structural conservation among different flaviviruses despite the poor sequence similarity. It has been reported that the flavivirus C protein has a high density of positive charge. These properties on structure and charge are critical for its affinity to both RNA and DNA [50,51,52]. The monomer of C protein is composed of four α-helices connected by short loops. The α1, α2, and α4 helices are in three different layers, and the α3 helix is a short helix that connects two C protein monomers on one side to form a C protein dimer [53,54]. A study which investigated the crystal structure of the C protein of WNV found that C protein was generally in the form of a symmetrical dimer in solution (Figure 2a). There exists a hydrophobic pocket formed by Leu29, Leu36, Phe45, Leu49, and Phe52 from α1 and α2 helicases of the dimer, connecting with the membrane (Figure 2b). The N-terminal of the C protein dimer forms a tetramer face-to-face with positively charged surfaces. Additionally, there are many positive residues which distribute in the C protein of WNV. These positive residues interact with RNA based on the nonuniform charge distribution [35,55]. Before virus assembly, the hydrophobic structure of the C-terminal of C protein in the cytoplasmic side of the endoplasmic reticulum (ER) is cleaved by NS2B-NS3 protease, and the C protein matures, containing approximately 100 amino acid residues [56,57].

C protein determines virus infectivity, protects the viral genome, and participates in the formation of the virus envelope and the maintenance of the E protein spatial structure. In the studies of TBFV and WNV, it is found that the NS2B/NS3 cleavage site is between the C protein and prM protein. After the hydrolysis in the Golgi body, the virus changes from an inert virus particle to an infectious one [56,58]. The discovery of the existence of C protein in the nucleus of cells infected by flavivirus reveals another functional possibility of C protein, that is, C protein can assist in mediating the transfer of antigens from the cytoplasm to the nucleus [57]. The interaction between C protein and the host nucleoprotein has also been observed. C protein can induce cytotoxicity in WNV-infected cells and arrest cell cycle at the G2 phase. These effects suggest the flexibility of C protein and may provide multiple ideal targets for the development of vaccines [57].

### 2.2. PrM and E Proteins

In immature flavivirus particles, parts of the prM protein are located at the tip of E protein, covering the DII region of three E proteins of each spike. When viral particles are transported into the Golgi body, the prM protein can avoid the premature fusion of E protein through covering fusion peptides. There are 60 icosahedral spikes composed of prM-E heterodimers on the surface of immature flavivirus particles. The prM-E heterodimers make the virus surface rough and protuberant. When the virus reaches the Golgi body, the N-terminal of prM protein is cleaved by furin; the prM protein turns into pr peptide and M protein in the process of virus maturation. As a partner of E protein, the prM protein can maintain the correct folding and secretion of E protein. It also facilitates the stability of E protein at low pH [58,59]. The important connection between prM protein and E protein is often used as the theoretical basis for the development of recombinant vaccines and other new vaccines, such as CYD-TDV for DENV, ChinZIKV for ZIKV, etc. In the mature state of flavivirus, the homodimers of E proteins are arranged into 30 rafts. Each raft contains three parallel dimers arranged on the outer surface of the virus in a herringbone pattern. Thus, the virus obtains a smooth surface and becomes fusogenic and infectious [60,61]. However, a recent report shows that the structures of DENV and ZIKV, unlike conventional flaviviruses, are temperature-dependent and have high structural plasticity. At elevated temperatures (e.g., 37 °C), the club-shaped particle is inducted, and a non-spherical structure containing a cylindrical tail and a disc-like head is obtained [62]. Thus, temperature-dependent viruses give us a reminder to compare the differences in the structure of normal viruses with those at different temperatures, and perhaps this key analysis has the potential to help us solve the problems associated with flavivirus infection.

At the entering step, the E protein of flavivirus contacts with its receptor. Initially, E protein is glycosylated at three amino acid sites: Asn130, Asn175, and Asn207. The glycosylated E protein contains two transmembrane helices which interact with other adhesion factors for increasing the virus density on the cell surface, thus facilitating the binding of the virus [59,63]. Similar to DENV, the E protein of WNV is the main structural component of the viral surface. Cryo-electron microscopy (Cryo-EM) shows that the envelope of the mature virus consists of 180 E glycoproteins in the form of a herringbone pattern. The envelope glycoprotein contains three domains (DI, DII, and DIII) which are connected to each other by flexible hinges to facilitate rearrangement and conformational change (Figure 2c).

DI is an eight-stranded β-barrel located at the N-terminal and is the central domain of the E protein. DI plays a critical role in stabilizing protein, and acts as a bridge-hinge between DII and DIII. The DII domain contains a hydrophobic fusion peptide that participates in membrane fusion. The discontinuous peptide forms a hinge in the n-octyl-β-D-glucoside (β-OG) region between DI and DII, and the hinge is mainly conserved in glycine and can trigger conformational changes which is the basis of virus maturation. This information may provide some novel ideas in dealing with virus infection. For example, MI-1148 is a furin inhibitor and can exert an effect on the β-OG domain where furin protease acts [64]. The DIII domain contains approximately 100 amino acid residues and has a β-barrel shape composed of six anti-parallel β-strands, and is the putative receptor-binding domain of E protein, that mainly interacts with cofactors and receptors. DIII stretches out of the virion surface as an apophysis, which can be recognized by neutralizing antibodies. Thus, DIII can be used as an antigen for serologic diagnosis and as the main target of neutralizing antibodies [65]. E protein belongs to type II fusion proteins and can mediate receptor binding and membrane fusion by rearranging a portion of the transmembrane segment (Figure 2c) [53,61,66,67]. A barrier for vaccine design is that the structures of some flaviviruses are affected by temperature. This effect makes the virus surface bumpy, thereby leading to looser arrangement of E protein. These characteristics may help the immune escape of flavivirus, and complicate vaccine development and drug design [62].

### 2.3. NS1 Protein

**Figure 3:**
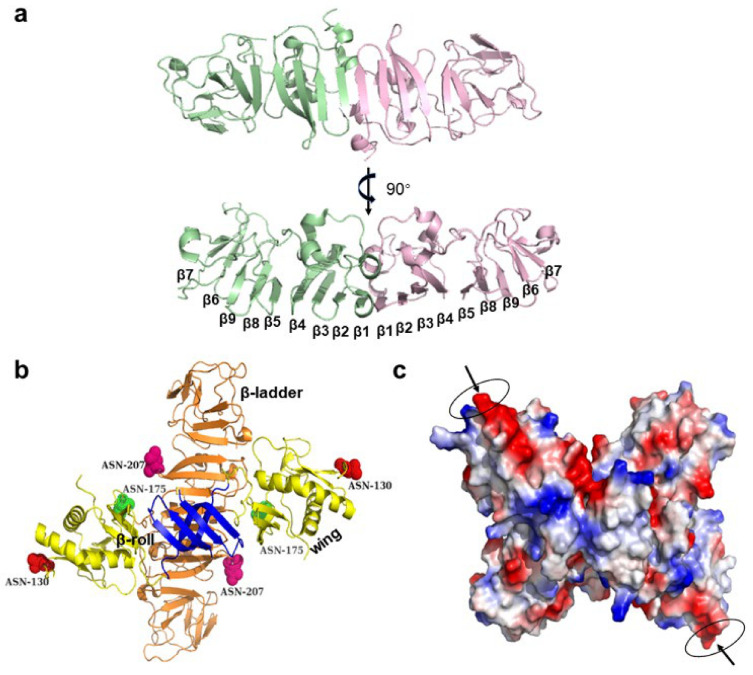
The structure of non-structural protein 1 (NS1). (**a**) The dimer structure of the NS1 protein of Zika virus (ZIKV). Two NS1 monomers are combined in the head-to-head form to produce dimers of NS1, and the β chain is extended and tiled into a larger plane (Protein Data Bank [PDB]: 5IY3 ZIKV). (**b**) The domains of the NS1 dimer protein of West Nile virus (WNV). NS1 protein contains three structural units, namely β-roll, β-ladder, and wing, which are, respectively indicated in blue, orange, and yellow; each monomer has three glycosylation sites (red): asn130, asn175, and asn207 (PDB: 4O6C WNV). (**c**) An octapeptide sequence in the C-terminal of NS1 plays an important role in cleavage; thus, it can be used as a target of antiviral therapy. Arrows indicate potential therapeutic targets (PDB: 4O6C WNV).

Among the non-structural proteins of flavivirus, NS1 (a highly conserved glycoprotein) has been detected in different intracellular positions of infected cells and in various oligomer forms, including monomer, dimer, and hexamer [68,69,70,71]. It is known that high degree of virus glycosylation is associated with high virulence. Therefore, some compounds, such as NN-DNJ, are designed to inhibit the glycosylation of NS1, thereby reducing the toxicity of NS1 secretion [72,73]. The monomer of NS1 has nine β-sheets flanked by a series of connecting loops and six pairs of cysteines at the C-terminal that form intracellular disulfide bonds in ZIKV (Figure 3a). The three-dimensional structure shows that the monomer of NS1 has three domains: small β-roll, “wing”, and “β-ladder” (Figure 3b) [70]. The “wing” domain is stretched from the central β domain resembling a “wing”, and contains two carbohydrate sites (Asn130 and Asn175) and an internal disulfide (Cys55-Cys143) for linking “wing” and “β-roll” [74,75]. The “β-ladder” domain is comprised of 18 β strands, where each monomer offers nine antiparallel rungs to form a ladder [75,76].

Following the synthesis of NS1 protein in cells, the NS1 dimer is formed and transported to the plasma membrane with the C-terminals in a head-to-head form. In the Golgi apparatus of infected cells, the NS1 dimer is sheared by glucosidase and glycosyltransferase to remove complex sugar; subsequently, the NS1 becomes soluble, secreting the hexamer [68]. The hexamer structure of NS1 shows that the “β-roll” faces to the inner side with a high hydrophobic bond, and the spaghetti loops, carbohydrate sites, and “wing” domain exist on the outer side. The three dimers form a wide and hydrophobic channel, which contains some hydrophobic amino acids (e.g., Ile183, Ile184, Gly185, Ala187, Leu206, and Trp210), and some aromatic residues (e.g., Phe8, Try122, Phe123, and Phe163) [70,74]. In WNV, the channel formed by the NS1 hexamer can be filled with lipid cargo, associating NS1 with flavivirus infection. The center of the NS1 hexamer can accommodate the host lipids to form a lipoprotein. Moreover, an octapeptide sequence in the C-terminal of NS1 plays an important role in cleavage and can be used as a target of antiviral therapy (Figure 3c).

NS1 triggers the release of cytokines, binds Toll-like receptors and endothelial glycogen enzyme, interferes with the normal function of the vascular system, and directly causes vascular leakage, which probably explained why NS1 of DENV is associated with severe vascular damage [74,77]. The NS1 antibody cross reacts with endothelial cells to induce apoptosis, which reveals endothelial dysfunction. In addition, NS1 is plentifully secreted into the circulatory blood in its hexamer form, and the circulatory NS1 can bind mannose-binding lectin and activate complement to achieve immune escape [78].

### 2.4. NS3 Proteins

**Figure 4:**
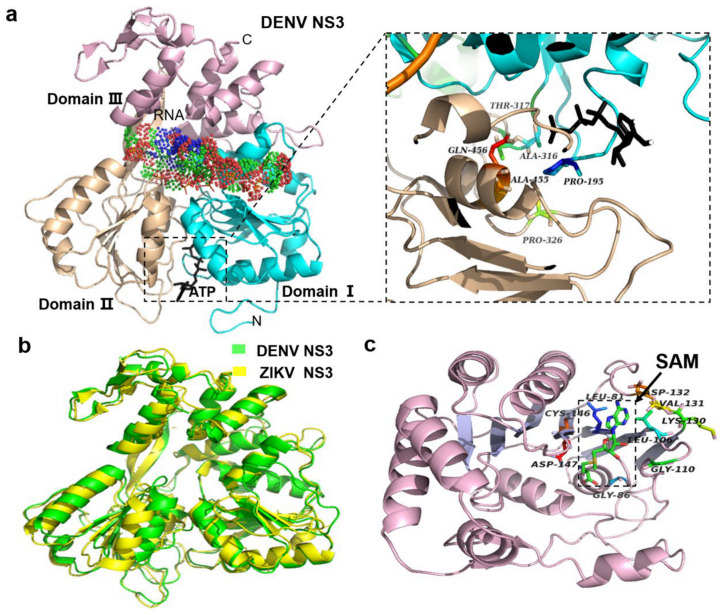
The structures of non-structural protein 3 (NS3) and NS5 of flaviviruses. (**a**) NS3 protein structure of dengue virus (DENV; Protein Data Bank [PDB]: 2JLV). The gap between the three domains (indicated by three different colors) is the RNA-binding site, and the adenosine triphosphate (ATP)-binding site is between domains I and II (see the enlargement of the framed area). (**b**) NS3 of Zika virus (ZIKV) and DENV. The root mean square (RMS) of the two viruses is 1.43, indicating that they are very similar. According to this reason, some proteins of other flaviviruses are also similar, which is very helpful for structural development and vaccine design (PDB: 2JLR DENV; PDB: 5JRZ ZIKV). (**c**) In the methyltransferase (MTase) core of NS5, there is a binding site of S-adenosylmethionine (SAM) (green in the framed area, indicated by an arrow). This site is also termed AdoMet, and plays an important role in the RNA capping process. This site is wrapped in the hydrophobic pocket of the central split (PDB: 2WA2 NKV).

NS3 contains two domains, the N-terminal protease domain encoding serine protease and the C-terminal helicase domain encoding RNA helicase/nucleoside triphosphatase (Figure 4a) [79]. These two domains are coupled by a short flexible linker. NS3 can cleave polyprotein into functional proteins, but the activity of NS3 needs to be activated by the 47 amino acid residues of NS2B, with a glycine link between NS2B and NS3 [3,80]. The NS2B–NS3pro structures of WNV and DENV show that NS2B acts as the cofactor of NS3. The C-terminal of NS2B forms a β-hairpin, and the tip of the β-hairpin is inserted into the active site of NS3 to activate it [81]. This structural basis provides a help for the subsequent development of related drugs. For example, the compound 3, as described in a later section, is an allosteric inhibitor which hinders the interaction between NS2B and NS3 [64,82]. The most obvious structure of NS3 is the chymotrypsin-like fold with two β-barrels. The His–Asp–Ser catalytic triad between the two barrels ZIKV NS3 is conserved. This catalytic triad could recognize its substrate with high specificity (Figure 4b) [83]. Furthermore, the C-terminal of NS3, as an RNA helicase, can break down double-stranded RNA (dsRNA) to provide a single-stranded RNA (ssRNA) template for the replication of the viral genome, and subsequently mediate the synthesis of the N terminal cap structure by RTPase. The energy of this capping process is provided by NTPase-hydrolyzed nucleotide [79]. NS3-helicase belongs to the superfamily two helicases, and has three domains. Domains I and II are similar and conserved, and may be related to nucleotide triphosphate hydrolysis. Domains I and II include an open sheet topology (Rossman fold) formed by six β-strands and three helicases. In the structure of DENV, ATP is located at the bottom of the gap. Domain III contains five similar parallel α-helicases and two reverse parallel β-strands (Figure 4a) [84]. In this gap, ST-610, an inhibitor of furin, was designed to prevent ATP hydrolysis [85]. In addition to contacting with RNA, NS3-helicase also interacts with NS5 of RNA-dependent RNA polymerase (RdRp) to complete capping [53,86].

### 2.5. NS5 Protein

NS5 is the largest (900 amino acids) and the most conserved protein in flavivirus. It is mainly composed of the N-terminal methyltransferase (MTase) and the C-terminal RNA dependent RNA polymerase (RdRp) [46]. MTase (1–265 amino acids) is a compact spherical folded monomer. The N terminal of MTase consists of a helix-turn-helix motif, followed by an α-helix and a β-strand. The C-terminal of MTase consists of an α-helix and a β-chain [87,88]. The core of MTase contains Rossmann folding, and the core domain provides binding sites for S-adenosylmethionine (SAM). In addition, the catalytic tetrads (Lys61, Asp146, Lys182, and Glu218) participate in the generation of CAP-1 as shown in NKV (Figure 4c). SAM is located at the binding pocket for S-adenosyl-L-homocysteine to contribute a methyl group to N-7 and 2′-O positions of virus cap methylations [58]. The adenine ring in the hydrophobic pocket is composed of Leu105, Val132, Phe133, and Ile147, and is stabilized by a hydrogen bond with main chain N of Asp131 and Val132 [89]. This information may extend our understanding on the target and aid future inhibitor design. Some compounds are designed to inhibit the function of SAM in viral replication, such as sinefungin, an analog of SAM which can occupy RNA binding sites [90].

RdRp contains three domains which resemble the right hand and six conserved motifs. The three domains include: (1) the characteristic fingers, “fingertips”, which contain a long insert of several strands; (2) the palm domain, which has four β-sheets flanking by three α-helices; (3) the thumb, which forms a polypeptide chain in the C-terminal. There exists a central template binding channel which is in the inner surfaces of the three domains. The motifs and domains could work together to control the volume of the template binding channel. This function ensures the combination of ssRNA with the active site in the template binding channel.

Guanylyltransferase transfers guanosine monophosphate into the genome of the new generation virus and methylates the cap structure at the corresponding position. Subsequently, through complex transformation, the new RNA can be correctly synthesized and output under the effect of RdRp [91,92]. To effectively translate viral multi proteins, the co-translation modification of mRNA and methylated Gpppn can protect mRNA from the interference of exonuclease [93]. This characteristic provides new idea for drug design. As described above, several proteins undergo conformational changes at the period of viral maturation. These changes may potentially offer some targets for developing flavivirus inhibitors.

## 3. Vaccines of Flavivirus

In many parts of the world, humans are suffered from flavivirus infections. The development of vaccines is the most hopeful strategy in the fight against virus epidemics. Vaccination is also the most basic measure in disease prevention. In the preparation of traditional flavivirus vaccines, such as live attenuated vaccines and inactivated vaccines, have indeed provided great help in solving flavivirus outbreaks in some areas. For example, the 17D vaccine of YFV and SA14-14-2 vaccine of JEV as live attenuated vaccines, and FSME-IMMUN^®^ and Encepur^®^ as inactivated vaccines. However, currently, vaccines are still scarce for other infectious flavivirus, and even those vaccines currently in use are not as effective and safe as expected in some situations due to mutations and variations of the viruses. Therefore, there is an urgent need to develop effective vaccines based on a comprehensive understanding of the structures and activity characteristics of viruses. Here, we introduce some vaccines which were designed based on certain important structural characteristics of flavivirus. The design concept of these vaccines is exactly what we want to emphasize in this review in order to benefit future vaccine design.

### 3.1. Live Attenuated Vaccines

Live attenuated vaccines are one of the most successful interventions in the history of human fight against viral diseases. Live attenuated vaccines which stimulate long-term immune protection have the potential to function as effective vaccines against flavivirus [94]. Considering effectiveness and cost, live attenuated vaccines have great advantages, but the safety issues of such vaccines need to be carefully considered. For example, it is easy to cause serious side effects after vaccination and mutations can restore the virulence of viruses in the human body [95]. Therefore, attention needs to be paid to the promotion of live attenuated vaccines. Some live attenuated vaccines that are still in clinical trials have been developed. TV003, a four full-length DENV serotype vaccine, shows significant antibody responses in a single injection [96,97]. The safety and immunogenicity of DENVax were demonstrated in phase I trials after the weakened DENV2 genotype was chimeric with prM and E proteins of other three serotypes [98,99]. Additionally, ZIKV live attenuated vaccine also uses a similar strategy to incorporate ZIKV prM and E proteins into the attenuated skeleton of DENV2 [100]. Although the candidate vaccines we mentioned above are optimized and updated, they are still in clinical trials. Among the live attenuated vaccines, 17D vaccine and SA14-14-2 vaccine have been successfully applied in clinical practice.

#### 3.1.1. 17D Vaccine

The 17D vaccine has been developed and used for the treatment of YFV infection. It was the first live attenuated vaccine for flavivirus which was developed in the 1930s. The 17D vaccine strain was obtained from the wild-type strain Asibi by serial passage in the tissue of chicken. This vaccine is proven to be safe and effective [101]. The effectiveness of 17D vaccine is due to its ability to effectively induce innate and adaptive immune responses in the body, resulting in the production of neutralizing antibodies against envelope proteins (E protein). In addition, 17D vaccine also regulates the balance of anti-inflammatory and pro-inflammatory cytokines in the body [102]. The commercialization of this vaccine helped control YFV infections and facilitated the development of other kinds of YFV vaccines [103]. The attenuated YFV strain 17D (YF17D) has been used to prevent YFV infection for decades. A single immunization can provide a lifelong immune response. In the 2016 YF outbreak in Angola and the Democratic Republic of Congo, over 18 million people were inoculated with 17D vaccine to prevent YFV infection [7]. Although the vaccine is considered as a huge success, immunocompromised people could suffer from serious complications after vaccination. YFV is still seen as a serious public health concern because of the constant sporadic YFV infection cases in recent years [104]. Based on the efficacy of YF17D as well as the maneuverability of its recombinant chimeric viral vector, YF17D has been used in the research of viral vectors of the YFV family (e.g., DENV gene vaccine and tumor gene therapy) in recent years [105].

#### 3.1.2. SA14-14-2 Vaccine

The SA14-14-2 vaccine, another vaccine obtained empirically, was developed for the prevention of JEV infection. The SA14-14-2 vaccine is the most widely used JE vaccine in the world [106]. This vaccine was obtained by several passages of virulent JEV SA14 strain on hamster kidney PHK cells and chick embryo PCE cells. In the mutation selection, results showed that the mutation G244E near the fusion domain of E protein weakened the damaging effect of JEV on the nervous system [107]. Moreover, the results of the studies of the full-length gene sequences of SA14-14-2 demonstrated its high genetic and phenotypic stabilities. The neurovirulence of this vaccine is relatively low and will remain largely unchanged [108]. The establishment of genetic and attenuated neurovirulence characteristics and the stabilities of SA14-14-2 virus are important in relation to vaccine safety in humans. In terms of cellular immunity responses, JEV SA14-14-2 is immunogenic for T cell IFNγ, which makes T cell responses frequent after vaccination [109]. This successful candidate vaccine is different from conventional live attenuated vaccines that simply target the entire viral genome and reminds us that mutation of the virus genome that encodes the key amino acids by sequence comparison in advance is important for the development of an effective virus vaccine.

### 3.2. Inactivated Vaccines

Inactivated vaccines are antigenic substances composed of inactivated material from a pathogen, such as a virus or bacterium. The viruses are cultured and subsequently inactivated through treatment with heating or chemicals (usually formalin). This kind of vaccine is relatively safe to use and simple to produce, but the immune effect is relatively low and the immune response time is short. Inactivated vaccines can be considered to act on the virus within a certain period, but once the mutation produces a new virus, its host specificity or virulence will change.

Relevant studies have shown that full-virus formalin inactivated vaccines are both safe and effective in the treatment of infection caused by TBEV. Two vaccines, FSME-IMMUN^®^ (Pfizer, New York, NY, USA) and Encepur^®^ (GlaxoSmithKline, London, UK), are based on the principle of using formalin-inactivated whole virus as antigen [110]. These vaccines are expected to induce an immune response against the E protein of the virus. However, viral non-structural protein was detected in the immunization investigation of the vaccinated population using mass spectrometry (MS) except proteins of the whole virus particle. Additionally, further studies have shown that the induction of NS1 specific antibodies may enhance the protective effect of the TBEV vaccine [111]. Unlike traditional TBEV inactivated vaccines, a new type of inactivated lyophilized vaccine Evervac has begun to come out. Vero cell culture, a popular cell substrate, was successfully used to produce inactivated polio vaccine. After adding 0.02% formaldehyde into the cell culture medium, the virus in the culture medium was inactivated, concentrated, and purified through the ultrafiltration method. In addition, it differs from the available vaccines, Evervac vaccine is absence of aluminum hydroxide adjuvant. Animal experiments have shown that the application of this vaccine can significantly increase the geometric mean titer (GMT) of anti-TBEV antibodies in serum, and this vaccine has been proven to be safe and effective in the phase II of clinical study [112]. These valuable attempts suggest that factors of both pathogens and hosts should be evaluated in vaccine development. TDENV-PIV is a tetravalent purified inactivated virus. After being produced in insect cells, it is inactivated with formalin and is currently in the phase I of experiments in many countries. ZPIV, a candidate human vaccine for ZIKV, is now tested in phase I preclinical studies. Similarly, the safety and immunity of the vaccine have also been proved [100].

### 3.3. Molecularly Engineered Vaccines

Genetic recombination approaches have been used in vaccine production to overcome the limitations of traditional vaccines (i.e., reduced immunogenicity, safety, and prolonged immune time). The design of engineered vaccines is based on the use of a gene engineering method or molecular cloning technology to isolate the protective antigen gene of a pathogen, and the transfer of the gene into a prokaryotic or eukaryotic system to express the protective antigen to produce the vaccine. An alternative way is to delete related gene(s) of the pathogen to weaken virulence. These two engineering routes may be used in the development of new vaccines which requires a full understanding of the virus structures. The following are some types of molecularly engineered vaccines.

#### 3.3.1. Recombinant Vaccine

The key to the success of the 17D vaccine is its ability to stimulate immune response in vivo through the expression of prM and E proteins. Due to the low production cost and well-established manufacturing process of 17D, it is used as a vector to develop other new vaccines of flavivirus. As flaviviruses have the same genome and replication characteristics, live attenuated DENV vaccine CYD-TDV, also named Dengvaxia, has been designed. This vaccine is a tetravalent vaccine developed by Sanofi Pasteur based on 17D vaccine vector backbone in which the genes encoding YFV structural proteins prM and E have been replaced by the corresponding genes of DENV-1,2,3, or 4. The live attenuated DENV vaccine CYD-TDV could induce an immune response in vivo by recombinant chimeras [113,114,115]. Results of phase III trials have demonstrated that the dengue vaccine did work well in people with previous exposure to DENV in the indicated population (age: ≥9 years). However, there are such major flaws in the implementation of this vaccine that some governments (e.g., Philippines) have sued the vaccine manufacturer (Sanofi) due to the excess deaths caused by this vaccine. In this condition, a new vaccine prototype is proposed for WNV, ChimeriVax-WN02, in which the prM and E proteins isolated from Flamingo strains are replaced by the 17D [94]. Moreover, amino acid mutations related to neurotoxicity (e.g., I107F, A316V, and K440R) in the JEV SA14-14-2 vaccine are added. Thus, the risk of the vaccine is reduced and the safety of the application is improved [107]. In addition, the recombinant chimeric ZIKV vaccine candidate (known as chinZIKV) can induce the expression of the prM-E protein of ZIKV using YFV SA 14-14-2 as the backbone. In animal experiments, it was found that a single dose can produce a strong and lasting immune response. In addition, it was observed that the vaccine exerted a protective effect on the fetus during pregnancy in mice. At present, more validations are warranted for human experiments [116,117]. For DENV, V180 is a tetravalent recombinant subunit protein vaccine and could induce high titers of antibodies in animal studies [100]. The success of these currently effective vaccines depends on the structure and the resolution of some important amino acids within the structure.

#### 3.3.2. Molecularly Cloned Vaccine

Considering the safety of vaccination, known viruses can be used as vectors to design chimeric viruses expressing structural proteins of flavivirus. Examples of virus vectors include the adenovirus vector, Venezuelan equine encephalitis virus vector, attenuated measles virus, and other safe and effective virus vectors. The E protein of the WNV strain is chimerized into the measles virus, and the secreted E protein of WNV and anti-WNV antibody have been detected in animal experiments [118]. According to this approach, the E proteins of the four types of DENV can also be encoded on the M protein domains and cloned into the measles virus [107]. It was found that this cloning method can enhance the immune response and produce four antibodies. In addition, the ZIKV vaccine was designed using an adenovirus vector that can stimulate the expression of E protein which was fused to a multiplication domain and produce immunoglobulin G in vivo, which may protect the fetus through the placental barrier [119].

Similar to this design concept, the VLP (virus-like particles) vaccine has a multiprotein structure that mimics the organization and conformation of authentic native viruses, but lack genomic RNA or DNA. VLPs have become a new impetus for vaccine development due to their excellent immune properties, excellent ability to induce innate and adaptive immune responses, and efficient and cost-effective safety templates. At present, hepatitis B and human papillomavirus vaccines are designed and developed through this approach. VLP provides a great basic support for the development of vaccines against diffident viruses [120]. In the new strategy of vaccine development against ZIKV, VLP is composed of three structural proteins and two non-structural proteins (NS2B/NS3). This VLP is continuously cultured in mammalian suspension cells and can self-assemble into particles, similar to ZIKV. This new VLP vaccine against ZIKV induces a high titer of immunity in vivo, showing its effectiveness [121]. In addition to maintaining the native epitopes structures, VLP vaccines can also reduce cross-reactions by modifying certain genes. VLP also designed for four DENV serotypes by co-expressing prM and E protein, and F108A mutation was introduced into the fusion loop of the E protein to decrease the antibody-dependent enhancement (ADE) [120]. All things considered, VLP does seem to be a preferable candidate for developing vaccines, but the safety of this type of vaccine still needs to be experimentally proven. Unlike conventional vaccines, the new type of vaccines is diversified by adding more non-structural proteins. Meanwhile, the functions and structural characteristics of these non-structural proteins should be better understood and appropriately applied to vaccine development.

#### 3.3.3. DNA Vaccine

The DNA vaccine is also termed the naked vaccine, because it does not need any chemical carrier. DNA vaccination is a technique to prevent diseases by injecting genetically engineered plasmids that contain DNA sequences encoding antigens, so the cells directly produce antigens, thereby causing a protective immune response. This vaccine platform is extremely attractive due to easy manufacture, ambient temperatures without the need for a cold chain, and ability to mimic natural infections.

In the development of a DNA vaccine for DENV by cloning prM and E gene inserts in the plasmid vector, recombinant plasmid gene encoding antigens are introduced into cells (APCs) to associate with MHC class I molecules and induce protective cytotoxic immune response. Unfortunately, the antibody titers are low and the immune response is not as active as expected. It was found that vaxfectin, a lipid-based adjuvant, could enhance immunogenicity for titers and provided protection against a DENV challenge [122]. In the development of the WNV vaccine, the plasmids encoding WNV prM and E protein were introduced into the expression system. This expression system can produce subviral particles which cause host immunity. However, the transfer efficiency of the expression structure is not high. Hence, the expression of plasmid genes needs to be improved. Similarly, the design of ZIKV vaccine can also use the DNA vaccine prototype as a backbone in the plasmids to encode prM and E proteins of ZIKV, yielding high titer antibodies in vivo. GLS-5700 was produced by this way [119]. The study found that anti-NS1 antibodies has cross reactivity with host molecules, so the antibodies can either be protective or deleterious to the host. At present, some molecules and amino acids which may help to design NS1 vaccine lacking cross-reactive anti-NS1 and preventing ADE are fined [123]. Furthermore, interferon (IFN) produced by host immune cells can coordinate the immune response together with other transcription factors, and enhance host immune response to viral infection. Studies have shown that the NS proteins of flavivirus can suppress the host antiviral immune response. Expression of DENV NS2A and NS4A/B can lead to downregulation of interferon (IFN)-β expression [124]. Then, N-terminal 125 amino acids of NS4B of DENV were found responsible for blocking IFN signal. Similar phenomena were also found in WNV and JEV. Mutation or knockout of those amino acids which inhibit IFN can be introduced into cDNA clone vaccines to produce attenuated viruses which can replicate but cannot inhibit IFN [125].

#### 3.3.4. mRNA Vaccine

mRNA vaccine is dramatically attractive in the face of the epidemic challenge. Considering the side effects caused by traditional vaccines, the emergence of mRNA vaccines may solve some problems that cannot be dealt with at present, and create more possibilities for the design of flavivirus vaccines in the future.

IgEsig-prM-E-LNP, an mRNA vaccine, was synthesized by chemically containing modified nucleoside 1-methylpseudouridine, 5′ and 3′ untranslated regions with poly-A, human IgE (the signal sequence IgEsig), and nucleoside-modified ZIKV full-length prM-E genes, and was encapsulated in lipid nanoparticles (LNPs) [126]. This vaccine was applied to mice infected with ZIKV, and could effectively induce an immune response [127]. Another advantage of mRNA is that it can modify genes. As we know, the main problem in the production of DENV vaccine is the difficulty in solving the ADE reaction. As a result, the candidate vaccines modify the E gene. In the mutation of the E gene, the fusion loop (FL) epitopes of domain II (DII) could reduce or eliminate cross-reactive antibodies. This strategy is a candidate for designing mRNA vaccines against ZIKV, DENV, and possibly other viruses.

The above-introduced vaccines have been produced and are in clinical trials. These vaccines mainly focus on the key proteins of flavivirus (Table 1). This situation puts forward higher requirements for understanding the protein structures of flavivirus. The key proteins are designed into pseudovirus, DNA or mRNA, and transferred into the body. Then, the corresponding antibodies and immune response work together to protect the body. At the same time, some key amino acids are mutated in these new vaccines to reduce side effects. We also pointed out that some vaccines still face unsolved problems, which pose great challenges to the development of safe and effective spectrum vaccines. For example, some vaccines have side effects after injection, clone vaccines face low transfection efficiency, difficulties in the selection of molecular adjuvants, and antibody dependent enhancement (ADE) et al. However, in addition to traditional vaccine production, increasing platforms for vaccine production have emerged with tremendous prospects for the prevention and control of flavivirus epidemics.

## 4. Structure-Based Anti-Flavivirus Drug Targets

Each of the 10 proteins of flavivirus plays a pivotal role in the life cycle of the virus, including the processes of virus entry, replication, assembly, and maturation. In the whole process, inhibiting mutation could weaken the replication of the virus. An alternative and complementary antiviral strategy is that vaccine design may also target the host cells except for focusing on the virus itself. The advantages of this approach include an increased threshold to the emergence of resistance and the possibility to target multiple viruses. Therefore, it is possible to develop anti-flavivirus drugs based on the characteristics and roles of the proteins in the life cycle of the virus and the structures of viral proteins. Following are some flavivirus proteins which show potential as antiviral drug targets based on their structures.

### 4.1. E Protein as an Antiviral Drug Target

**Figure 5:**
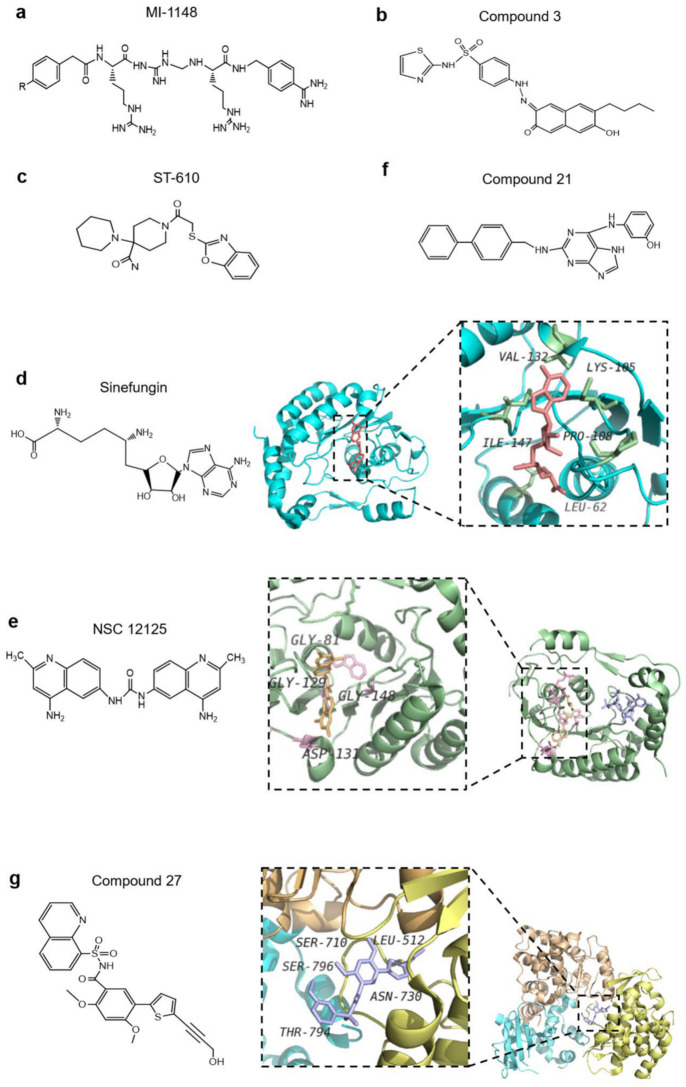
Anti-flavivirus compounds. (**a**) 4-(guanidinomethyl)-phenylacetyl-Arg-Tle-Arg-4-aminoboenzylamide (MI-1148) is a furin protease inhibitor which blocks the cracking of furin and weakens the toxicity of viral protein. (**b**) Compound 3 is an allosteric inhibitor which blocks the interaction between non-structural protein 2B (NS2B) and NS3. (**c**) ST-610 prevents adenosine triphosphate (ATP) hydrolysis in the cell culture of dengue virus (DENV). (**d**) Sinefungin is a natural product that potently inhibits the activity of flavivirus; however, like S-adenosylmethionine (SAM), it can produce cytotoxicity. The amino acids of flavivirus that interact with sinefungin are shown in the structure (PDB: 5KQS). (**e**) NSC12155 inhibits SAM by binding to the SAM cofactor site of the methyltransferase (MTase) (PDB: 5CUQ). (**f**) Compound 21 is a 2,6-diaminopurine derivative which inhibits the NS5 RNA-dependent RNA polymerase (RdRp) of DENV. (**g**) Compound 27 binds to the allosteric site of RdRp and inhibits the replication of dengue virus type 2 (DENV2) (Protein Data Bank [PDB]: 5K5M).

Based on the understanding of the three regions of E protein and the dynamic processes of protein–protein interaction, there exist plenty of potential sites of action for drugs that can be developed to inhibit viral infection. E glycoprotein has three domains. These three domains are connected by a polypeptide linker. The hinge motion of the polypeptide linker plays a crucial role in the rearrangement of E protein when virus transforming during immature, mature and fusion. A ligand-binding capsule exists at the hinge between the DI and DII domains of E protein. It was observed in the crystal structure of DENV-2 that N-octyl-β-D-glucoside (β-OG) located at a hydrophobic region between DI and DII of the E protein monomers. The nearby loop (ki) controls pocket deformation by movement, where the opening of the pocket can bind the β-OG region, and the ki loop and ij loop near the hydrophobic region form a salt bridge and hydrogen bond to assist the migration of the DII fusion peptide to the host membrane and promote fusion. Therefore, designing a small molecule that can combine with the pocket to form more hydrogen bonds may cause a conformational change of E protein before it reaches the Golgi apparatus [128], thus destroying the synthesis of virus particles and E-mediated membrane fusion, for example, cyanohydrazones inhibit 3-110-22 and JBJ-01-162-04 [129]. Prior to flavivirus particles reaching the Golgi body, prM and E proteins are cleaved by furin protease in host cells, and the virus particles mature. At present, a developed polybasic compound, 4-(guanidinomethyl)-phenylacetyl-Arg-Tle-Arg-4-aminoboenzylamide (MI-1148), is a furin protease inhibitor (Figure 5a), which blocks the cracking of furin and weakens the toxicity of viral protein [130]. This inhibitor has the highest potency but a mouse study revealed that the inhibitor had just a limited therapeutic range for the virus. According to this design idea, furin inhibitors may be developed and used to block flavivirus maturation in the future, by optimizing some key amino acids. Furthermore, low pH triggers the rearrangement of E-glycoprotein, and allows the virus to mature and be released into the serum. Based on this mechanism, researchers may design some drugs to make the environment alkaline and destroy the maturation of virus. Chloroquine, designed according to this concept, can alkalize organelles in antimalarial drug therapy [131]. Based on these discoveries, it is rational to hypothesize that drugs which alkalize the internal environment of cells could block the rearrangement of E-protein, making the virus unable to become infectious. Similarly, based on the thought of defending flavivirus from entering the host, design of inhibitory peptides for the stem-specific conserved sequence of E protein to form viral membrane pores may neutralize the virus genome by the host cell in advance. Curdlan sulfate, a small molecule designed according to the mechanism of high hexaacid heparin sulfate, can act on the cell receptor of E protein and host target protein, and effectively prevent the entry of virus. Hence, it can effectively control the ADE phenomenon [85].

### 4.2. Non-Structural Proteins as Antiviral Drug Targets

#### 4.2.1. NS1 Protein

NS1 is a conserved glycoprotein. It is known that high degrees of virus glycosylation are associated with high virulence. Two conserved N-linked glycosylation sites at N130 and N207 were discovered in the NS1 of DENV. Related studies have shown that when these two glycosylation sites are mutated, the secretion of NS1 to serum is reduced and its function becomes unstable [72]. Therefore, these two glycosylation sites of NS1 are potential targets and may provide new ideas for drug design. For example, (n-Nonyl)-deoxygalactonojirimycin (NN-DNJ), an iminosaccharide derivative, can significantly reduce the secretion of NS1 [72,73]. Meanwhile, WNV and MVEV inhibit the dimerization of NS1 through mutant residue 250, thus inhibiting the replication of the mutant virus [107]. In the Golgi apparatus of infected cells, the complex sugar is removed from NS1 dimer, subsequently, the NS1 becomes the hexamer. The middle of the hexamer structure of the NS1 protein is a hydrophobic channel with “β-roll” facing to the inner side with a high hydrophobic bond, which is rich in lipids, and may be more convenient to infect the host cell. In recent years, studies have made great progress regarding the hydrophobic channel and lipid-directed antiviral targets. Nicotinic acid (a triglyceride synthesis inhibitor) can improve lipid concentration in the microenvironment of the replication complex and reduce the secretion of NS1 through the action of diacylglycerol acyltransferase 2. As with nicotinic acid, methyl-β-cyclodextrin (a cholesterol isolation compound) could also improve the lipid concentration in the microenvironment of the replication complex and reduce the secretion of NS1 [73].

#### 4.2.2. NS3 Protein

Viral proteases are involved in the replication of RNA, virion assembly, and immune escape. Therefore, the proteases are considered essential antiviral drug targets. NS3 protease, a trypsin-like serine protease, is one of the most important proteases in flavivirus, and plays an important role in genome replication and protein folding. Meanwhile, a short peptide of NS2B is an important auxiliary sequence for NS3 protease. There are three important amino acids on the active site of NS3, namely, serine, histidine, and aspartic acid [132]. High-throughput screening (HTS) has been used to screen the substrate peptides which may modify the residues of the active sites, thereby potentially inhibiting the flavivirus. Inhibitors of substrate peptides designed by (HTS) have been tested in WNV infection. Compound 3 is an allosteric inhibitor with limited cytotoxicity and can block the interaction between NS2B and NS3 (Figure 5b) [64,82]. NS3 helicase opens the hydrogen bond between the two chains. One approach for designing drugs to inhibit the function of the helix is to destroy the activity of ATPase and block the energy supply. It is found that the ST-610 inhibitor meets this criteria for preventing ATP hydrolysis in the cell culture of DENV (Figure 5c) [85]. Another approach is to prevent the binding of flavivirus with nucleic acids. A short peptide containing a protease-cutting site has been designed to competitively inhibit the activity of NS3 protease, potentially nullifying the helix lysis function of NS3 [125]. Ivermectin, a broadly anti-helminthic drug, targets the viral helicase and is proved to be an inhibitor of YFV replication [133]. A third approach is to design a short peptidase as inhibitory ligand to cover certain specific sites of flavivirus protein, such as bivalent inhibitors containing a lysine-head and arginine-head. These inhibitors can occupy specific binding sites of NS3, thus hindering the replication of virus genes [134].

#### 4.2.3. NS5 Protein

MTase of the N-terminal and the RdRp functional domains of the C-terminal of NS5 is crucial for genome stability, efficient translation, and escape from immune response, and thus are considered potential therapeutic targets [89,135,136]. MTase is a compact spherical folded monomer, its core domain provides binding sites for S-adenosylmethionine (SAM) which participates in flavivirus RNA capping pathway [137]. Sinefungin, a natural product, similar to the role of SAM, exhibits potent inhibitory activity for impeding both N7 and 2′-O-methylation reactions (Figure 5d) [138,139]. It is worth noting that the cellular non-permeability and non-selectivity of these inhibitors are limited [90]. The structural characterization further showed a conserved hydrophobic pocket near the SAM, which also offers more possibilities to design inhibitors [139]. It was found that 4-fluorophenyl, a SAM analog, blocked RNA methylation and inhibited virus replication via occupying binding sites for the base and the 2′OH groups of cap-0-adenosine [140,141]. NSC12155 is also an MTase inhibitor which binds to the SAM cofactor site of the MTase to perform an inhibitory function on WNV, DENV-2, and JEV (Figure 5e) [142,143]. It could be even better if the specificity problems of these inhibitors are solved. Through the screening of a compound library, (guanine-N7)-methyltransferase (N7MTase) and (nucleoside-2′-O-)-methyltransferase (2′OMTase) were found to combine with the specific active sites of MTase, thus interfering with the activity of MTase and incapacitating MTase to complete the capping as well [134,144]. All these effects impair the gene replication of the virus. NS5-RdRp was considered as a major drug target for its potential advantage of virus unique activity which confers way to the development of inhibitors with fewer side effects [145]. The C-terminal of RdRp contains three domains which control the volume of the template binding channel to ensure that ssRNA can enter the active site, where the allosteric pocket of the starting ring near the RdRp can also be used to design small nucleoside inhibitors targeting allosteric regulation for the prevention of virus replication [140], which would prove to be a promising treatment approach. The pocket is close to the active site; the designed compound 27 can bound to this allosteric pocket and inhibit the replication of DENV2 (Figure 5f) [146]. Sofosbuvir is a nucleotide polymerase inhibitor used in clinics to against hepatitis C virus which is distantly related with ZIKV [147]. Relevant studies of cell culture and animal have also proved that sofosbuvir inhibits viral genome replication in ZIKV infection [148]. Furthermore, early treatment of sofosbuvir increased the survival rate of ZIKV-infected animals, and sofosbuvir was also found to prevent the acute neuromotor impairment. FDA approved this drug to anti-ZIKV as an inhibitor of NS5B polymerase nucleoside [149]. Likewise, 2′-C-methyladenosine (2′CMA) and 7-deaza-2′-C-methyl adenosine (7DMA) also exhibited high potency against flaviviruses. Through high-throughput technology screening, some compounds are identified as RdRp inhibitors, and their structures are synthesized in optimization [150]. Compound 21 is a 2,6-diaminopurine derivative which modifies quinoline and 26-diaminopurine scaffolds and possesses activity against three serotypes of DENV (Figure 5g) [125]. Furthermore, the interactions between NS5 and NS3 are still a breakthrough framework for the development of antiviral therapeutic strategies against flavivirus.

### 4.3. Other Inhibitors of Non-Structural Proteins of Flaviviruses

Studies on some flaviviruses indicate that NS protein can participate in the suppression of host IFN. In animal experiments, mutation or knockout of some amino acids of the designed cDNA vaccines can cause a strong host immune response. Based on this finding, we can design some drugs to resist these amino acids and to enhance the immune response against the virus. IFNα-2b and diethyldithiocarbamate (DDTC) are two potential compounds which are proved safe and are in clinical trials aiming to verify their efficacy. The former therapeutic compound reduced morbidity and the severity of disease symptoms, whereas the latter was able to cross the blood–brain barrier and to inhibit virus infection in the central nervous system and delay morbidity [125]. In addition, some drugs can synergistically enhance the efficacy of IFN and improve the safety and efficacy of IFN treatment. For example, ribavirin (a broad-spectrum RNA virus replication inhibitor) can combine with IFN and better control flavivirus infection, as observed in vitro [134,151]. Thus, the combination of broad-spectrum RNA inhibitors and IFN may be more effective for the treatment of viral infection. Furthermore, some compound drugs can weaken the virulence and immune escape ability of the virus, thus reducing the host immune barrier and enhancing immune response. For example, lovastatin can reduce the release of newly synthesized virus particles and further infection; acyclovir is more effective in the treatment of herpes virus infection; bortezomib has the ability to inhibit proteases in DENV and ZIKV [152]. These drugs fundamentally weaken the virulence and immune escape ability of the virus, thus reducing the host immune barrier, enhancing the immune response, and suppressing flaviviruses infection.

## 5. Discussion

Due to the absence of effective antiviral drugs and vaccines, repeated outbreaks of flavivirus are possible. People in most areas of the world have suffered from the epidemic of flavivirus, putting life safety, public health, and global economy in a dire state. Therefore, it is necessary for us to understand the epidemic characteristics and transmission routes of different flaviviruses in advance to produce an early warning. Analyses of the structural characteristics of the ten proteins of flavivirus provide great help in making breakthroughs in the design and development of more effective and safer broad-spectrum treatments.

In the previous designs of flavivirus vaccines and therapeutic targets, researchers focused their attention on the E protein of the viral envelope. However, experiments also find that the E protein-induced antibody has a limited protection range and has the risk of cross-reactions and antibodies with poor neutralization ability during the immune process. Recently, a study analyzed the complex structure of 1G5.3 and DENV or ZIKV, and found that monoclonal antibody 1G5.3 had a protective effect on a variety of flaviviruses [153]. This study reveals the mechanism of broad-spectrum protection of NS1 for the first time. Another study found that, in the co-crystal of NS1 protein of DENV-1 or DENV-2 serotypes, the protective antibody 2B7 can recognize a conserved epitope of the β-ladder at the C-terminal of NS1, and can effectively eliminate blood vessel leakage and reduce fatality rate [154]. To avoid the ADE effect, the non-structural protein NS1 of flavivirus has undoubtedly become a new target for vaccine design.

Interestingly, American scientist Michael Houghton and his colleagues determined the genetic sequence of a new virus in 1989 and named it hepatitis C virus (HCV). As the HCV genome is similar to flavivirus in structure and phenotypic characteristics, flavivirus is classified as HCV in the Hepacivirus genus of Flaviviridae family [155,156]. IFNα combined with ribavirin is also the standard regimen (SOC) for the treatment of chronic hepatitis C approved by EASL. Direct-acting antivirals (DAAs) are considered the ideal choice for the treatment of chronic HCV patients, and this treatment has been proved efficient and has minimal adverse effects [157]. The inhibitor of HCV protease, boceprevir (BOC) or telapi Wei (TVR), or a triple therapy with interferon and ribavirin, has been approved for clinical use in the United States in May 2011. The triple therapy is recommended for patients with HCV genotype 1, which can improve the cure rate. In addition, the NS3 structure of HCV had been determined and NS3 inhibitors were designed. Among these inhibitors, 3-heterocyclylquinolone is the most pungent inhibitor which also exhibited good hydrogen bond interactions with the modeled protein [158]. These treatments for HCV may be an important reminder for the treatment of flavivirus infection.

Even at the present global SARS-CoV-2 epidemics, we should be cautious and treat the potential next flavivirus epidemic as a high priority. Currently, accelerating the development of vaccines and anti-viral drugs are likely the key ways to solve the epidemic. Through joint efforts across the world, we may hopefully develop effective anti-flavivirus technologies in the near future.

## Figures and Tables

**Table 1:** The information of flavivirus vaccines.

Vaccine	Vaccine Type	Antigen	An-Virus	Stage
17D	Live attenuated vaccine	E protein	YFV	licensed
SA14-14-2	Live attenuated vaccine	E protein	JEV	licensed
TV003	Live attenuated vaccine	prM-E	DENV_1–4_	Phase III
DENVax	Live attenuated vaccine	prM-E	DENV_1–4_	Phase III
FSME-IMMUN	Inactivated vaccine	E protein	TBEV	licensed
Encepur	Inactivated vaccine	E protein	TBEV	licensed
Evervac	Inactivated vaccine	E protein	TBEV	Phase II
TDENV-PIV	Inactivated vaccine	C-prM-E-NS1/3/5	DENV_1–4_	Phase I
ZPIV	Inactivated vaccine	E protein	ZIKV	Phase I
CYD-TDV(Dengvaxia)	Recombinant vaccine	prM-E	DENV_1–4_	licensed
ChimeriVax-WN02	Recombinant vaccine e	prM-E	WNV	Phase II
V180	Recombinant vaccine	E protein	DENV_1–4_	Phase I
ZIKV-VLP	VLPs(Virus-like particles)	C-prM-E-NS2B/NS3	ZIKV	Animal
DENV-VLP	VLPs(Virus-like particles)	prM-E	DENV_1–4_	preclinical
GLS-5700	DNA vaccine	prM-E/NS1	ZIKV	Phase I
IgEsig-prM-E-LNP	mRNA vaccine	prM-E	ZIKV	Animal
